# Radiotherapy and subsequent thyroid cancer in German childhood cancer survivors: a nested case–control study

**DOI:** 10.1186/s13014-015-0521-6

**Published:** 2015-10-31

**Authors:** Isabelle Finke, Peter Scholz-Kreisel, Ulrike Hennewig, Maria Blettner, Claudia Spix

**Affiliations:** Institute of Medical Biostatistics, Epidemiology and Informatics, University Medical Centre of the Johannes Gutenberg University Mainz, Mainz, 55101 Germany; University Childrens’ Hospital Essen, Pediatric Haematology and Oncology, Essen, 45147 Germany; German Childhood Cancer Registry, Institute of Medical Biostatistics, Epidemiology and Informatics, University Medical Centre of the Johannes Gutenberg University Mainz, Mainz, 55101 Germany

**Keywords:** Thyroid cancer, Second malignant neoplasm, Childhood cancer survivor, Radiotherapy, Case–control study

## Abstract

**Background:**

Radiotherapy is associated with a risk of subsequent neoplasms (SN) in childhood cancer survivors. It has been shown that children’s thyroid glands are especially susceptible. The aim is to quantify the risk of a second neck neoplasm after primary cancer radiotherapy with emphasis on thyroid cancer.

**Methods:**

We performed a nested case–control study: 29 individuals, diagnosed with a solid SN in the neck region, including 17 with thyroid cancer, in 1980–2002 and 57 matched controls with single neoplasms were selected from the database of the German Childhood Cancer Registry. We investigated the risk associated with radiotherapy exposure given per body region, adjusted for chemotherapy.

**Results:**

16/17 (94.1 %) thyroid SN cases, 9/12 (75 %) other neck SN cases and 34/57 (59.6 %) controls received radiotherapy, with median doses of 27.8, 25 and 24 Gy, respectively. Radiotherapy exposure to the neck region increased the risk of the other neck SNs by 4.2 % (OR = 1.042/Gy (95 %-CI 0.980-1.109)) and of thyroid SN by 5.1 % (OR = 1.051/Gy (95 %-CI 0.984-1.123)), and radiotherapy to the neck or spine region increased the thyroid risk by 6.6 % (OR = 1.066/Gy (95 %-CI 1.010-1.125)). Chemotherapy was not a confounder. Exposure to other body regions was not associated with increased risk.

**Conclusions:**

Radiotherapy in the neck or spine region increases the risk of thyroid cancer, while neck exposure increases the risk of any other solid SN to a similar extent. Other studies showed a decreasing risk of subsequent thyroid cancer for very high doses; we cannot confirm this.

## Background

As a result of improved diagnostic procedures and progress in treatment in the past decades, about 81 % of childhood cancer patients nowadays survive at least 15 years [1]. As a consequence, the importance of late effects in the follow-up care of childhood cancer survivors has increased. One of the most severe late effects is the occurrence of a subsequent neoplasm (SN) [2, 3]. In Germany, currently 4.7 % of childhood cancer patients develop a second malignant neoplasm (SN) within twenty-five years of the first neoplasm [1]. Chemotherapy and radiotherapy (RT) have been shown to have a considerable impact on the risk of an SN [2, 4–6]. Besides leukaemia and breast cancer, thyroid cancer is most frequently associated with radiation exposure [2]. Gul et al. [7] identified high doses absorbed by the thyroid gland due to scattered radiation during radiotherapy for breast cancer, lung cancer, Hodgkin’s lymphoma and tumours in the head and neck region using CO^60^ teletherapy. An association between the occurrence of thyroid adenomas and the radiation dose received during childhood cancer treatment has been recently shown [8]. An update from the Childhood Cancer Survivor Study (CCSS) [5] demonstrated that children’s thyroid glands were particularly susceptible to radiation during cancer therapy. A pooled analysis of several epidemiological studies illustrated that the relative risk (RR) for thyroid cancer increases with increasing radiation dose, up to 15-fold at an estimated does of 10 Gy to the thyroid gland [6] compared to patients with no radiation therapy. This analysis also showed that the risk of thyroid cancer declines after high-dose radiotherapy, but it is assumed that elevated risk continues for many decades [6]. Based on these results, it is expected that long-term survivors have an increased risk of a subsequent thyroid cancer. Younger age at diagnosis, female sex and longer time since exposure to radiation were found to increase this effect [5], but a meta-analysis showed no modification of radiation effects by sex [6]. Furthermore it is questionable if the tissue of thyroid glands is specifically sensitive to the influence of radiation in comparison to the entire neck region.

In this study, we analysed the relationship between radiotherapy exposure of the neck region and the occurrence of cancer as a second solid tumour in relation to cumulative radiation dose per body region (given dose). The emphasis is on thyroid cancers; other cancers of the neck region are investigated to assess the specificity of the thyroid effect. The exposure data available are given as the dose per body region, which allows exposure region specificity to be investigated.

## Methods

### Study population

This paper presents a subgroup of the patients published in [9, 10], where the patient groups are described in detail. Briefly, the 33,809 patients with a childhood malignancy diagnosed from 1980 to 2000 registered in the German Childhood Cancer Registry (GCCR) in 2003 served as basis for a nested case–control study [9]. As a federal registry, case ascertainment by the GCCR pertains to all malignancies diagnosed in German residents up to 14 years of age and has achieved a high degree of completeness [1]. Patients are followed up indefinitely with an emphasis on complete registration of subsequent malignancies. SNs had been diagnosed before 06/30/2002 and at least three months later than the first neoplasm (FN). The controls were patients who had not experienced an SN by the date of selection and were matched by sex, age at diagnosis, year at diagnosis and follow-up time since FN. For the analysis presented here, the SNs located in the neck region were selected: 17  thyroid cases, 12 other second neoplasm cases, 57 controls. All individuals included in this study gave general informed consent at the time of being registered by the GCCR. At time of data retrieval, neither active participation nor a specific ethics approval was required, as only existing data from an approved data base were used.

### Therapy data

Information on therapy for the first cancer was available for all cases and controls from medical records. The body regions targeted by radiotherapy were classified into 10 regions [10]. A given dose is defined as the radiation dose in Gy applied to the respective body region. The cumulative dose to each body region was calculated as the sum of all given doses to this region. Absorbed doses for body regions, organs or tissues were not available.

### Statistical analysis

Statistical analysis was conducted using conditional logistic regression as appropriate for nested case control studies. We used the given radiation doses as a continuous variable, assuming a linear dose–response relationship with log(OR). The effects of dose by region were assessed as follows: The main model (Model 1) estimated the effect of the dose given to the neck region. To identify other relevant exposed regions, the respective cumulative radiotherapy doses to every single other body region were additionally included in the model, and the best fitting combination was selected with a backward selection procedure (cut off level p = 0.1) (Model 2). If more than one exposed region had an effect, a combined model was estimated (Model 3). Alternatively, the sums of the exposure to all other body regions (Model 4) or adjacent body regions of the neck (head, thorax and spine) only (Model 5) were included in the model in addition to the neck dose. Results are expressed as odds ratios (OR) per Gy with two-sided 95 % confidence intervals (CI).

A number of sensitivity analyses were performed: Chemotherapy was investigated as a potential confounder by including separate chemotherapeutical agent indicator variables in the model. Alternative shapes of the dose–response curve were tested according to Royston et al. [11]. Potential effect modification by year of diagnosis, age at diagnosis, latency time or sex was analysed by including categorised effect modifiers. Years (age, diagnosis, latency) were categorised in groups of 5 years.

## Results

The second malignant thyroid cancers in the 17 cases (10 males, 7 females) included papillary adenocarcinoma (n = 12), follicular adenocarcinoma (n = 4), and one unspecified adenocarcinoma. The 12 other second malignant cancers located in the neck region included Hodgkin’s lymphomas (n = 8), one non-Hodgkin’s lymphoma, one fibrosarcoma and two other specified soft tissue sarcomas (ICCC-3 IX(d)) (Table 1).

**Table 1:** Characteristics of cases with an SN in neck region by FN

		Thyroid SN					Any other neck SN (8 HL, 1 NHL, 3 STS)				
ICCC group	First neoplasm type	Cases n	RT+ n	Median age at diagnosis of FN (range)	Median age at diagnosis of SN (range)	Median time interval FN to SN in years (range)	Cases n	RT+ n	Median age at diagnosis of FN (range)	Median age at diagnosis of SN (range)	Median time interval FN to SN in years (range)
I	Leukaemias	8	8	4.5 (1–14)	15.5 (8–30)	11.5 (7.4-16.6)	7	5	4 (2–13)	8(4–17)	4 (1.6-6.1)
II	Lymphomas	5	4	10 (7–13)	19 (14–25)	7.3 (4.0-17.6)	3	3	12 (8–14)	20 (18–24)	12.5 (4.2-12.9)
III	CNS tumours	2	2	3.5 (2–5)	13 (11–15)	9.1 (8.9-9.3)	0	-	-	-	-
IV	Neuroblastomas	0	-	-	-	-	1	0	0 (−)	4 (−)	3.6 (−)
VI	Renal tumours	1	1	3 (−)	12 (−)	8.5 (−)	1	1	0 (−)	15 (−)	14.5 (−)
IX	Soft tissue sarcomas	1	1	1 (−)	13 (−)	11.5 (−)	0	-	-	-	-
Total	All FN	17	16	5 (1–14)	15 (8–30)	9.3 (4.0-17.6)	12	9	4.5 (0–14)	12 (4–24)	4.3 (1.6-14.5)

CNS: central nervous system

FN: first neoplasm

HL: Hodgkin’s Lymphoma

ICCC: International Classification of Childhood Cancer

NHL: Non-Hodgkin’s Lymphoma

RT+: radiotherapy received

SN: second neoplasm

STS: Soft Tissue Sarcoma

Characteristics of 17 cases with SN of the thyroid and 12 cases with any other neck SN diagnosed 1980–2002 by FN (ICCC-3 classification [1]) diagnosed in 1980–2000 in Germany, FN below the age of 15

SNs in thyroid cases occurred between the ages of 8 and 30 years, and in other SN cases between ages 4 and 24 years. The median interval between the diagnosis of the FN and the SN was 9.3 years (range 4.0-17.6 years) for thyroid cases and 4.3 years (range 1.6-14.5 years) for other cases (Table 1).

The most frequent FNs among all neck SN cases were leukaemias and lymphomas (Table 1), while most frequent FNs among the controls were leukaemias, lymphomas and renal tumours (Table 2).

**Table 2:** Characteristics of 57 matched controls

ICCC group	First cancer type	Controls n	RT+ n	Median age at diagnosis of FN (range)
I	Leukaemias	22	18	5 (1–14)
II	Lymphomas	12	7	9 (1–14)
III	CNS tumours	6	3	5.5 (4–13)
IV	Neuroblastoma	1	0	1 (−)
VI	Renal tumours	9	3	1 (1–12)
VII	Hepatic tumours	1	0	0 (−)
VIII	Bone tumours	1	1	3 (−)
IX	Soft tissue sarcomas	2	1	4.5 (4–5)
X	Germ cell tumours	2	0	1 (0–2)
	Other non-malignant neoplasm	1	1	14 (−)
Total	All FNs	57	34	5 (0–14)

CNS: central nervous system

FN: first neoplasm

ICCC: International Classification of Childhood Cancer

RT+: radiotherapy received

Characteristics of 57 matched controls by FN (ICCC-3 classification [1]) diagnosed in 1980–2000 in Germany, aged under 15 years

16 of 17 the thyroid SN cases (94.1 %), 9 of 12 other neck SN cases (75 %) and 34 of 57 controls (59.6 %) had received radiotherapy to at least one body region with a median cumulative dose over all body regions of 27.8, 25 and 24 Gy respectively (Table 3).

**Table 3:** Body regions exposed to radiotherapy and median cumulative exposure

	Thyroid SN		Any other neck SN (8 HL, 1 NHL, 3 STS)		Controls	
Body region	Exposed cases	Median cumulative exposure of exposed cases in Gy (min-max)	Exposed cases	Median cumulative exposure of exposed cases in Gy (min-max)	Exposed Controls	Median cumulative exposure of exposed controls in Gy (min-max)
Head	10	19 (18–55)	4	12 (12–30)	27	24 (12–110)
Neck	4	30.3 (25–35)	3	35 (30–35)	4	32.5 (20–40)
Spine	4	24 (18–35)	2	24 (−)	6	29.6 (15–40)
*Neck or spine* ^*a)*^	*8*	*25.3 (18–35)*	*5*	*30 (24–35)*	*10*	*30.1 (15–40)*
Thorax	2	25.3 (25–25.6)	2	35 (−)	4	30.5 (20–40)
Abdomen	1	30 (−)	1	25 (−)	7	25 (20–36)
Pelvis	1	32 (−)	0	-	0	-
Legs	0	-	0	-	0	-
Arms	0	-	0	-	0	-
Unexposed	1	-	3	-	23	-
Total exposed (Max exposure per person)	16	27.8 (18–55)	9	25 (12–35)	34	24 (12–110)

FN: first neoplasm

Gy: Gray

HL: Hodgkin’s Lymphoma

ICCC: International Classification of Childhood Cancer

NHL: Non-Hodgkin’s Lymphoma

SN: second neoplasm

STS: Soft Tissue Sarcoma

^a)^: Exposed individuals are either neck or spine exposed, never both

Exposed body regions of 29 cases with SN of the thyroid or any other neck SN diagnosed 1980–2002 and 57 matched controls by FN (ICCC-3 classification [1]) diagnosed in 1980–2000 in Germany, FN at age under 15 years

Four thyroid SN cases (23.5 %), 3 other neck SN cases (25 %) and 4 controls (7 %) had received radiotherapy to the neck region with respective median doses to the neck region of 30.3, 35 and 32.5 Gy, ranging from 20–40 Gy (Table 3); all these patients had Hodgkin’s Lymphoma as the FN. All 17 thyroid SN cases, all 12 of the other neck SN cases and 50 of the controls (87.7 %) were exposed to chemotherapy.

Radiotherapy exposure to the neck region increased the risk of thyroid cancer by OR _RT(Neck)_ = 1.051/Gy (95 %-CI 0.984-1.123) and other neck SNs OR _RT(Neck)_ = 1.042/Gy (95 %-CI 0.980-1.109) (Model 1, Table 4). This means that people exposed to 20 Gy had a 2.703-fold risk increase and a 4.51-fold risk increase at 30.3 Gy (median dose given at the neck) for a thyroid SN.

**Table 4:** Conditional logistic regression analysis

		Thyroid SN (17 cases, 34 controls)		Any other neck SNs (8 HL, 1 NHL, 3 STS) (12 cases, 23 controls)		Any neck SN (29 cases, 57 controls)	
Model		Odds ratio/1 Gy	95 % CI	Odds ratio/1 Gy	95 % CI	Odds ratio/1 Gy	95 % CI
1	RT dose in region of SN (neck)	1.051	0.984-1.123	1.042	0.980-1.109	1.046	1.000-1.095
2	RT dose in region of SN (neck)	1.057	0.992-1.127	1.042	0.979-1.109	1.051	1.004-1.101
	RT dose in region of spine	1.080	0.994-1.173	0.995	0.931-1.063	1.031	0.985-1.079
3	RT dose in region of spine or neck	1.066	1.010-1.125	1.019	0.974-1.067	1.041	1.006-1.078
4	RT dose in region of SN (neck)	1.048	0.980-1.120	1.045	0.981-1.113	1.046	1.000-1.095
	Cumulative RT dose in other body regions	1.004	0.983-1.027	0.988	0.949-1.029	1.000	0.982-1.019
5	RT dose in region of SN (neck)	1.045	0.979-1.116	1.043	0.980-1.110	1.045	0.999-1.093
	Cumulative RT dose in adjacent body regions^a^	1.009	0.986-1.034	0.994	0.948-1.042	1.006	0.986-1.027

CI: confidence interval

FN: first neoplasm

Gy: Gray

HL: Hodgkin’s Lymphoma

ICCC: International Classification of Childhood Cancer

NHL: Non-Hodgkin’s Lymphoma

RT: radiotherapy

SN: second neoplasm

STS: Soft Tissue Sarcoma

^a^head, thorax, spine

Results from conditional logistic regression analysis of effect of RT doses in the region of the neck, all other regions or at regions adjacent to the neck in 17 cases with SN of the thyroid and 12 cases of any other neck SN diagnosed 1980–2002 and 57 matched controls by FN (ICCC-3 classification [1]) diagnosed in 1980

Exploring the exposure of other body regions, a combination of neck and spine exposure is associated with thyroid cancer cases (OR _RT(Neck or spine)_ =1.066 (95 %-CI 1.010-1.125)) (Model 3, Table 4), while only the neck region exposure is relevant for other neck region SNs (Model 2, Table 4). The additional cumulative doses of all other or all adjacent body regions do not have an effect on thyroid or other neck region SNs (Model 4 and 5, Table 4).

The model for all second neck cancers was not improved by adding exposure to other body regions into the model.

**Fig. 1:**
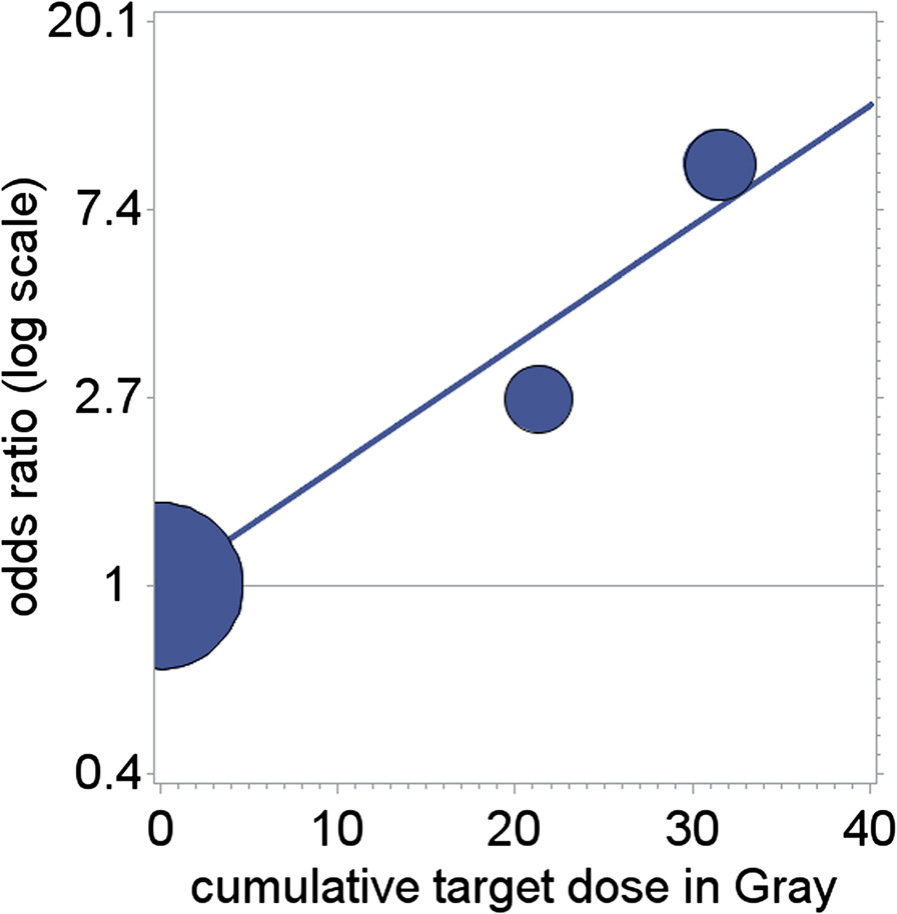
Dose–response relationship. Dose–response relationship odds ratio (OR) of thyroid SN and cumulative radiotherapy given dose in the neck or spine region in 51 German childhood cancer survivors (17 SN cases, 34 controls) first diagnosed in 1980–2000. The line is the regression result. The bubbles are OR estimates of grouped dose data for illustration purposes. Mean dose per dose group is given on the horizontal axis. Bubble sizes are proportional to the number of patients per dose group

Regarding the dose–response relationship between radiotherapy and risk of subsequent thyroid cancer, a linear relationship with the log(OR) could be seen in our study. Figure 1 shows the dose–response relationship from Model 3 for thyroid SNs. Other dose response curves were rejected in the sensitivity analysis.

There was no change in the effect of radiotherapy when including chemotherapy in the thyroid SN model; chemotherapy had no independent effect on the risk of a second thyroid or other neck region tumour either. Risk estimates were similar in all age groups, for male and female, for year of diagnosis and for latency years (data not shown). However, data are too sparse to investigate effect modification effects in depth.

## Discussion

Results from our small nested case–control study show that radiotherapy applied to the neck region increases the risk of a second tumour in the thyroid gland by 5.1 % per one Gy total given dose, and radiotherapy applied to the neck or spine region significantly increased the risk by 6.6 % per one Gy of the total dose given. Radiotherapy in the neck region had a similar, though slightly smaller, effect on the risk of other SNs in the neck region (4.2 %/Gy).

It must be noted that neither exposure to the thorax nor to the head had an effect on thyroid SNs (or other neck SNs) in addition to exposure to the neck, although most patients with exposure to the neck also had a head and/or thorax exposure due to their Hodgkin’s lymphoma treatment protocols. Most patients with spinal exposure were primary leukaemia patients. Radiotherapy given to regions other than the neck or spine did not seem to further increase the risk of SNs in the neck region. Confounding by chemotherapy was not present. The sample was too small to detect differences between subgroups.

We conclude that the risk of a second neoplasm in the neck region increases specifically with radiotherapy exposures in the neck (and spine) region but not with exposure to other body regions. Furthermore the effect is not entirely specific to thyroid tumours: the risk of other second tumours, such as lymphomas and sarcomas, is almost as high per dose given.

The results are not quite comparable to results of other studies on subsequent thyroid cancer after childhood cancer [5, 6, 12]. Bhatti et al. [5] described a non-monotonous relationship, with estimated absorbed thyroid doses of up to 60 Gy, peaking at 20 Gy. A previous analysis by Ronckers et al. [12] found a similar dose response curve. Veiga et al. [6] pooled data from US, UK, Nordic and Canadian studies (including the aforementioned study by Bhatti et al. [5]) and found a similar dose response curve. Fitted RR for 20 Gy in all three cited studies are about 13 to 15 fold compared to 2.7 fold in this study. However, the pooled study also shows that estimates in different study groups vary widely, and different dose response curves have a surprisingly similar fit. The sensitivity modelling did not show a preference in our data for a non-monotonous dose response relationship, including a peaked curve (see also Fig. 1). However, the number of exposed cases, particularly exposed cases with doses of 30 Gy or more, was basically too small to fit more complex models. It is difficult to compare given doses to absorbed doses; however, as an example, for Hodgkin Lymphoma patients Gul et al. [7] estimate that 64-94 % of the given dose is also absorbed by the thyroid.

The median time interval for the occurrence of second thyroid cancer was 9.3 years (Min 4.0 years, max 17.8 years) in this study. However, follow-up is somewhat shorter compared to other studies, which reported latency periods of 12.5 to 19 years [3, 5, 13–15].

Chemotherapy was not found to be a confounder for the association between radiotherapy and thyroid cancer risk, which is consistent with findings of Bhatti et al. [5] but inconsistent with Veiga et al. [6], who found an effect modification of chemotherapy for alkylating agents, anthracyclins and bleomycin.

While this study was too small to show effect modification by age, sex, year or latency, the previous study by Hennewig et al. [10], assessing all solid second tumours, showed a higher risk per radiotherapy dose for cases younger at first diagnosis, diagnosed earlier (1980’s as opposed to the 1990’s) and after a longer latency period, but not by sex. Age and latency as effect modifiers were also reported by other studies on subsequent thyroid cancer [3, 5]. Bhatti et al. [5] reported a higher risk of SN of the thyroid gland in women, in which they saw a reflection of the generally higher proportion of females in the population of thyroid cancer patients. The meta-analysis by Veiga et al. [6] showed no modification of radiation effects by sex.

Our study has several strengths and limitations. The main limitation of this study is the small number of cases. However, a notable risk increase could be shown nonetheless. The follow-up time of maximally 18 years of the cases was rather short; more subsequent thyroid cancer cases are expected from this cohort with further follow-up.

One of this study’s strengths is that it is an unselected cohort from a fairly complete population-based registry representative for the German population of childhood cancer patients [1]. All known cases with a malignant second neoplasm of the thyroid gland observed between 1980–2002 in Germany after a first childhood neoplasm were included, and their controls were chosen from the same database. The other strength is the recording of the dose data by body regions, which allows the contribution of the exposure of all body regions to the risk of thyroid SNs and other neck SNs to be assessed separately and jointly.

## Conclusion

Our results indicate that radiotherapy exposure of the neck or spine region in childhood can be related to the occurrence of thyroid cancer as an SN as early as 4 years later. For neck radiation exposure, the risk of other SNs in the neck region (lymphomas, sarcomas) occurring even earlier is almost as large as for second thyroid cancer. The sample was small; however, the study group is unselected and well defined. Childhood cancer survivors treated with radiotherapy to the neck and spine region should be followed up closely for SNs in the thyroid, despite the pros and cons of thyroid screening [16]. Other subsequent cancers may also occur in an irradiated neck region.

## Abbreviations

CCSS: Childhood cancer survivor study
CI: Confidence Interval
FN: First neoplasm
GCCR: German childhood cancer registry
Gy: Gray
Max: Maximum
Min: Minimum
n: Number
OR: Odds ratio
RR: Relative risk
RT: Radiotherapy
SN: Second or subsequent neoplasm
